# Integrating imaging and genomic data for the discovery of distinct glioblastoma subtypes: a joint learning approach

**DOI:** 10.1038/s41598-024-55072-y

**Published:** 2024-02-28

**Authors:** Jun Guo, Anahita Fathi Kazerooni, Erik Toorens, Hamed Akbari, Fanyang Yu, Chiharu Sako, Elizabeth Mamourian, Russell T. Shinohara, Constantinos Koumenis, Stephen J. Bagley, Jennifer J. D. Morrissette, Zev A. Binder, Steven Brem, Suyash Mohan, Robert A. Lustig, Donald M. O’Rourke, Tapan Ganguly, Spyridon Bakas, MacLean P. Nasrallah, Christos Davatzikos

**Affiliations:** 1 Center for Biomedical Image Computing and Analytics (CBICA), University of Pennsylvania, 3700 Hamilton Walk, 7Th Floor, Philadelphia, PA 19104 USA; 2 Center for AI and Data Science for Integrated Diagnostics, University of Pennsylvania, Philadelphia, USA; 3 Department of Radiology, Perelman School of Medicine, University of Pennsylvania, Philadelphia, PA USA; 4 Center for Data-Driven Discovery in Biomedicine (D3b), Division of Neurosurgery, Children’s Hospital of Philadelphia, Philadelphia, PA USA; 5 Department of Neurosurgery, Perelman School of Medicine, University of Pennsylvania, Philadelphia, PA USA; 6 Penn Genomic Analysis Core, Perelman School of Medicine, University of Pennsylvania, Philadelphia, PA USA; 7 Department of Bioengineering, School of Engineering, Santa Clara University, Santa Clara, CA USA; 8 Penn Statistics in Imaging and Visualization (PennSIVE) Center, Department of Biostatistics, Epidemiology, and Informatics, Perelman School of Medicine, University of Pennsylvania, Philadelphia, PA USA; 9 Department of Radiation Oncology, Perelman School of Medicine, University of Pennsylvania, Philadelphia, PA USA; 10 Abramson Cancer Center, Perelman School of Medicine, University of Pennsylvania, Philadelphia, PA USA; 11 Glioblastoma Translational Center of Excellence, Abramson Cancer Center, University of Pennsylvania, Philadelphia, PA USA; 12 Department of Pathology and Laboratory Medicine, Perelman School of Medicine, University of Pennsylvania, Philadelphia, PA USA; 13 Division of Computational Pathology, Department of Pathology & Laboratory Medicine, School of Medicine, Indiana University, Indianapolis, IN USA

**Keywords:** Cancer imaging, Machine learning

## Abstract

Glioblastoma is a highly heterogeneous disease, with variations observed at both phenotypical and molecular levels. Personalized therapies would be facilitated by non-invasive in vivo approaches for characterizing this heterogeneity. In this study, we developed unsupervised joint machine learning between radiomic and genomic data, thereby identifying distinct glioblastoma subtypes. A retrospective cohort of 571 IDH-wildtype glioblastoma patients were included in the study, and pre-operative multi-parametric MRI scans and targeted next-generation sequencing (NGS) data were collected. L21-norm minimization was used to select a subset of 12 radiomic features from the MRI scans, and 13 key driver genes from the five main signal pathways most affected in glioblastoma were selected from the genomic data. Subtypes were identified using a joint learning approach called Anchor-based Partial Multi-modal Clustering on both radiomic and genomic modalities. Kaplan–Meier analysis identified three distinct glioblastoma subtypes: high-risk, medium-risk, and low-risk, based on overall survival outcome (*p* < 0.05, log-rank test; Hazard Ratio = 1.64, 95% CI 1.17–2.31, Cox proportional hazard model on high-risk and low-risk subtypes). The three subtypes displayed different phenotypical and molecular characteristics in terms of imaging histogram, co-occurrence of genes, and correlation between the two modalities. Our findings demonstrate the synergistic value of integrated radiomic signatures and molecular characteristics for glioblastoma subtyping. Joint learning on both modalities can aid in better understanding the molecular basis of phenotypical signatures of glioblastoma, and provide insights into the biological underpinnings of tumor formation and progression.

## Introduction

Glioblastoma is the most common and aggressive form of primary brain cancer in adults. Despite advances in treatment, the survival rate for glioblastoma patients remains low, with a median survival time of only 15 months^1,2^ using conventional standard of care. Treating glioblastoma is particularly challenging due to its heterogeneous nature^3^, with patients exhibiting diverse clinical, imaging, and molecular features.

To enhance the understanding of glioblastoma's heterogeneity and improve treatment outcomes, researchers have endeavored to subtype this disease into more homogeneous groups based on a range of biological and clinical characteristics. Previous efforts for glioblastoma subtyping have concentrated on imaging subtyping^4–8^ and molecular subtyping^9–13^. Imaging subtyping uses magnetic resonance imaging (MRI) to categorize glioblastoma into different subtypes based on their imaging characteristics, such as tumor size and location. Molecular subtyping uses gene expression data to categorize glioblastoma into different subtypes based on their molecular profile. These approaches provide insights into the underlying biology of the disease and help identify new therapy targets.

The complementary strengths of these subtyping methods suggest that jointly leveraging the information provided by both types of data to subtype glioblastoma may yield even more biologically and clinically relevant subgrouping^14–16^. Therefore, the aim of this study is to design a joint learning approach to explore the integration of multiple sources of data, specifically multi-parametric MRI and genomics, to uncover reproducible dominant dimensions spanning the heterogeneity of glioblastoma across both phenotype and genotype. This could offer a unique opportunity to discover non-invasive biomarkers and enhance decision support models.

## Materials and methods

### Study design

**Figure 1:**
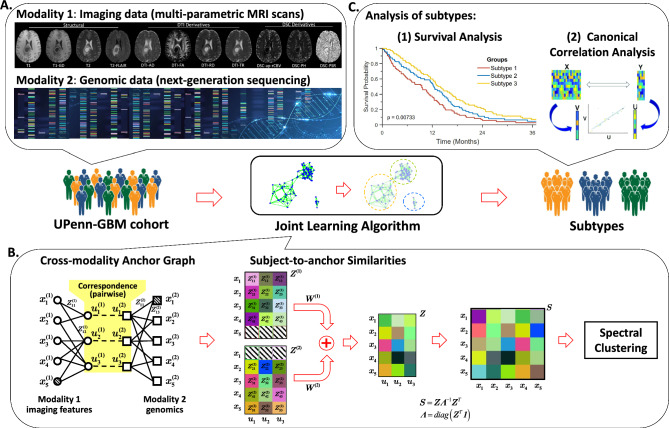
The study workflow. (**A**) The first modality of glioblastoma data is multi-parametric MRI (T1, T1-Gd, T2, T2-FLAIR, DSC, DTI) scans. The second modality is targeted next-generation sequencing (NGS) based genomic data. (**B**) Anchor-based Partial Multi-modal Clustering, a joint learning approach is applied to both modalities. (**C**) Analysis of the identified glioblastoma subtypes, *e.g.*, survival analysis and canonical correlation analysis.

The study is comprised of three main modules, as depicted in Fig. 1. The first module focuses on data acquisition and processing, which includes extracting radiomic imaging features and genomic data (for a detailed description, please refer to section "Data collection and preprocessing"). The second module aims to apply a joint learning approach to both modalities to identify glioblastoma subtypes. Cluster membership of each patient is obtained as a result of this module, providing an indicator of the patient's subtype. The third module concerns the analysis of subtypes, which involves the analyses on overall survival (OS) of different subtypes. In addition, statistical analysis is performed to characterize the imaging and genomic data of each subtype. To quantify the relationship between the two modalities, the well-known multi-variate analysis method—Canonical Correlation Analysis (CCA)^17–19^ is employed.

### Data collection and preprocessing

The institutional review board (IRB) at the University of Pennsylvania (UPenn) approved all experimental protocols, and all methods were conducted in compliance with relevant guidelines and regulations. Our study was fully compliant with the health insurance portability and accountability act (HIPAA). The IRB of UPenn granted a waiver of informed consent for this study and all patients had previously given informed consent for participation in research studies. We retrospectively collected data from a subset of 571 IDH-wildtype adult patients from the UPenn-GBM cohort^20^. Exclusion criteria for patients included: (1) incomplete MRI scans with at least one sequence missing; (2) detection of IDH mutation; or (3) treatment received outside of the University of Pennsylvania Health System.

*MRI acquisition.* Pre-operative multi-parametric magnetic resonance imaging (mpMRI) scans were obtained on a 3 Tesla scanner. The scans consisted of T1-weighted (T1), T1 with contrast-enhanced (T1-Gd), T2-weighted (T2), T2 fluid-attenuated inversion recovery (T2-FLAIR), diffusion tensor imaging (DTI), and dynamic susceptibility contrast-enhanced (DSC)-MRI sequences. The details about image acquisition parameters can be found in the reference^20^.

*Image preprocessing.* The mpMRI sequences were re-oriented to left-posterior-superior (LPS) coordinate system, co-registered and resampled to resolution of 1 mm^3^ using Greedy Algorithm^21^, and skull-stripped using CaPTk^22^ version 1.9.0 (www.med.upenn.edu/cbica/captk/) for brain extraction. Tumors were segmented into the enhancing tumor (ET), non-enhancing core (NC), and peritumoral edema (ED) subregions from mpMRI scans, using CaPTk tumor segmentation module.

*Imaging features.* To extract radiomic features from the three tumor subregions, *i.e.*, ET, NC, and ED, we employed the above mentioned CaPTk, which yielded a series of features. These radiomic features were extracted with feature categories of first-order intensity-based statistics, histogram, volumetric, gray-level co-occurrence matrix (GLCM), gray-level run length matrix (GLRLM), gray-level size-zone matrix (GLSZM), neighborhood gray-tone difference matrix (NGTDM), and Collage features. All extracted features were normalized using z-scoring before further analyses. The features were further post-processed by removing dimensions with small standard deviation (SD, σ ≤ 1E-6) and those with high correlation (r ≥ 0.85). The final cohort for further analysis comprised 462 IDH-wildtype patients with a total of 971 imaging features.

*Genomic data.* The tumor samples obtained from the patients in this cohort were subjected to sequencing using one of the two targeted next-generation sequencing (NGS) panels developed in-house. The details of these panels are described in the reference^23,24^. This study employed a total of 27 genes, which were common in two utilized sequencing panels and thus, considered for analysis. These genes include *ARID2, ATRX, BRAF, CDKN2A, CIC, DNMT3A, EGFR, FGFR2, FUBP1, IDH1, IDH2, KDR, KRAS, MDM4, MET, NF1, NOTCH2, NTRK1, PDGFRA, PIK3CA, PIK3R1, PTEN, PTPN11, RB1, SETD2, SMARCB1, TP53*. Patients with mutations in *IDH1* or *IDH2* were excluded from the study. Ultimately, the genomic data of 355 IDH-wildtype patients with 25 genes were included in the cohort for further analysis.

### Feature selection and discovery-replication split

It is widely recognized that genes do not act in isolation but instead interact to carry out various biological functions^25^. In this study, we took into account the glioblastoma pathways outlined in this seminal research paper^25^, including *RB1* pathway (including *RB1* and *CDKN2A* genes), *P53* pathway (including *MDM4* and *TP53* genes), *MAPK* pathway (including *BRAF* and *NF1* genes), *PI3K* pathway (including *PTEN, PIK3R1, PIK3CA* genes), and *RTK* pathway (with *FGFR2, MET, PDGFRA, EGFR* genes). We used these 13 genes from 5 different signaling pathways for our joint learning approach.

Considering the high dimensionality and redundancy among the acquired imaging features, we employed an L21-norm based feature selection method^26^, which could identify a more informative subset of features from the original 971 dimensions. In order to further improve the integration of the imaging and genomic data, we utilized the pathway mutation information as a supervised label to guide the feature selection process. This allowed us to prioritize imaging features that were most relevant to the specific pathways implicated in the genomic data.

To avoid ambiguity, we denote the matrix of imaging features as X and the matrix of gene pathway mutation information as Y, without loss of generality. Each column of matrix X represents the imaging features of a patient, while each column of matrix Y represents the gene pathway mutation information of a patient. The objective function of feature selection is

1 $$\mathop {\min }\limits_{W} \left\| W \right\|_{{21}} \,\, s.t.W^{T} X = Y,$$

where the L21 norm measures the sum of the magnitudes of the L2 norms of the rows of feature selection matrix W. The resulting output W from this method exhibits row-wise sparsity, where any row that consists entirely of zeros (or small magnitudes < 1E-6) indicates the imaging feature dimension that should be filtered out. Leave-one-out cross validation was performed to determine the optimal number of selected imaging features. After evaluating different numbers of features, we ultimately selected 12 imaging features.

The included patients were divided equally into a discovery cohort and a replication cohort. Table 1 provides an overview of the characteristics of the patients in both cohorts.

**Table 1:** Patient Demographics (n = 571).

Characteristics	Discovery cohort	Replication cohort
No. of patients, n (%)	285	286
With imaging and genomic data	131 (45.96)	115 (40.21)
With imaging data only	94 (32.98)	122 (42.66)
With genomic data only	60 (21.06)	49 (17.13)
Age, n (%)		
> 65	122 (42.81)	145 (50.70)
≤ 65	163 (57.19)	141 (49.30)
Mean ± SD	62.97 ± 11.56	64.49 ± 11.57
Sex, n (%)		
Male	171 (60.00)	177 (61.89)
Female	114 (40.00)	109 (38.11)
*MGMT* methylation, n (%)		
Methylated	71 (24.92)	54 (18.88)
Unmethylated	108 (37.89)	93 (32.52)
Indeterminate or not available (N/A)	106 (37.19)	139 (48.60)
EOR, n (%)		
Near/Gross total resection	141 (49.47)	172 (60.14)
Partial resection or biopsy	106 (37.19)	93 (32.52)
Not available (N/A)	38 (13.34)	21 (7.34)
Survival months, mean ± SD	15.63 ± 15.58	13.98 ± 11.39

### Joint learning approach

As shown in Table 1, the patient data in each modality has incompleteness, *i.e.*, some subjects may only have data available in one modality (imaging or genomics). To address this issue, we utilized Anchor-based Partial Multi-modal Clustering^27^, a joint learning approach that considers both modalities together. We constructed anchor graphs^28^ to connect all patients' modalities, and established stationary Markov random walks over the graph. The one-step and two-step transition probabilities were calculated to serve as similarities. Finally, we performed Spectral Clustering^29^ on the fused similarity matrix to obtain a unified clustering result. The optimal number of clusters was determined by gap statistic^30^.

Figure 1B illustrates the joint learning approach utilized in this study. The shaded circle/square means that the subject's corresponding modality is actually missing. The incompleteness in each modality was well addressed by *K* pairs of anchor points {*u*_*i*_^(1)^,*u*_*i*_^(2)^}_*i*=1,2,…,*K*_ which were selected from the common subjects existing in both modalities. In each modality, the subject-to-anchor similarity Z_*ij*_ between the existing subject *x*_*i*_ and anchor point *u*_*j*_ was calculated by Gaussian function^28^. Stationary Markov random walks were established over each modality's bipartite graph. The one-step transition probabilities *p*^<1>^ were calculated by Z_*ij*_ as follows:

2 $${p}^{\langle 1\rangle }\left({x}_{i}|{u}_{j}\right)=\frac{{{\text{Z}}}_{ij}}{\sum_{i{\prime}}{{\text{Z}}}_{i{\prime}j}},$$

3 $${p}^{\langle 1\rangle }\left({u}_{j}|{x}_{i}\right)=\frac{{{\text{Z}}}_{ij}}{\sum_{j{\prime}}{{\text{Z}}}_{ij{\prime}}}={{\text{Z}}}_{ij}.$$

The two-step transition probabilities *p*^<2>^ serve as similarities {S_*ij*_}, which are calculated as

4 $${{\text{S}}}_{ij}={p}^{\langle 2\rangle }\left({x}_{i}|{x}_{j}\right)={p}^{\langle 2\rangle }\left({x}_{j}|{x}_{i}\right)=\sum_{k}{p}^{\langle 1\rangle }\left({x}_{i}|{u}_{k}\right){p}^{\langle 1\rangle }\left({u}_{k}|{x}_{i}\right)=\sum_{k}\frac{{{\text{Z}}}_{ik}{{\text{Z}}}_{jk}}{\sum_{i{\prime}}{{\text{Z}}}_{i{\prime}k}}.$$

The similarity matrix S, ultimately obtained, served as the input for the Spectral Clustering^29^ algorithm, with the resulting clustering membership indicating the subtypes.

It is worth noting that our approach follows a transductive learning framework, which allows for reasoning from observed cases to specific cases. This approach is particularly well-suited for small datasets, where learning a general rule is challenging due to limited samples.

### Statistical analysis

We conducted statistical analysis using R software version 4.2.2 (http://www.R-project.org) and MATLAB R2021a (https://www.mathworks.com), as appropriate. The Kruskal–Wallis test was used for continuous variables, and the Fisher's exact test was used for categorical variables when comparing differences in clinical, radiomic, and molecular features across subtypes in each cohort. To compare the Kaplan–Meier survival curves, we used the log-rank test. We evaluated statistical significance of the survival curves of subtypes using a Cox proportional hazards model at a 95% confidence interval.

### Canonical correlation analysis (CCA)

Hotelling^17–19^ originally defined CCA as a statistical method aimed at extracting the common information between two data tables that measure quantitative variables on the same set of observations. CCA calculates two sets of linear combinations, known as latent variables, for each data table to maximize correlation. In this study, we employed CCA to investigate the association between imaging and genomic data.

Specifically, we utilized the 246 patients who had both imaging and genomic data available. The 12-dimensional imaging features for each subject were represented as X, while the corresponding 13-dimensional genomic data were represented as Y. Each row of X and Y corresponded to a specific subject. In CCA, the problem is to find two latent variables, denoted *u* and *v* obtained as linear combinations of the columns of, respectively, X and Y. The coefficients of these linear combinations are stored, respectively, in the vectors *a* and *b*; and, so, we are looking for *u* = X*a* and *v* = Y*b* satisfying

5 $${\text{r}}=\underset{a,b}{\mathrm{arg max}}\frac{{u}^{T}v}{\sqrt{\left({u}^{T}u\right)\left({v}^{T}v\right)}}.$$

The variable r is the canonical correlation between latent variables *u* and *v*, which is maximized by CCA. The solution of CCA is obtained through matrix eigen-decomposition.

## Results

### Survival analysis of subtypes

**Figure 2:**
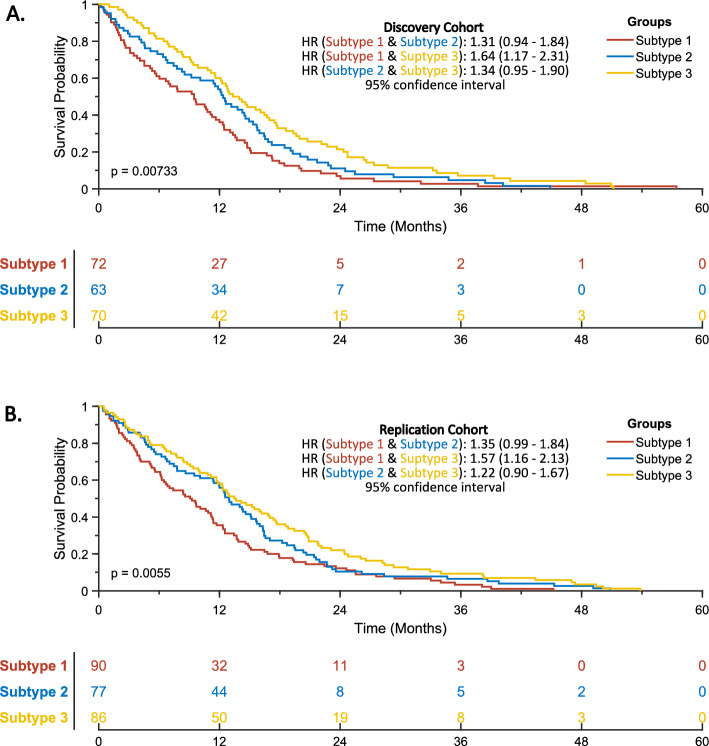
Kaplan–Meier survival curves of the identified subtypes on (**A**) discovery and (**B**) replication cohorts.

Our joint learning algorithm produced three clusters, each representing a distinct subtype, with significant differences in overall survival rates among the subtypes (*p* < 0.05 using Log-Rank (Mantel-Cox)). Specifically, the identified subtypes were high-risk, medium-risk, and low-risk. Kaplan–Meier curves were generated based on clinical information, and revealed that the high-risk subtype 1 had the worst survival outcome, followed by the medium-risk subtype 2 and low-risk subtype 3. In the discovery cohort (Fig. 2A), the hazard ratio (HR) between subtypes 1 and 2 was 1.31 (0.94–1.84), between subtypes 1 and 3 was 1.64 (1.17–2.31), and between subtypes 2 and 3 was 1.34 (0.95–1.90). Similar survival tendencies were observed in the replication cohort (Fig. 2B), where the HR between subtypes 1 and 2 was 1.35 (0.99–1.83), between subtypes 1 and 3 was 1.57 (1.16–2.13), and between subtypes 2 and 3 was 1.22 (0.90–1.67).

To assess the impact of various factors on OS, we utilized the Cox Proportional Hazards Regression^31^ model (Cox). Specifically, we applied Cox to examine the effects of known factors in survival such as age, O^6^-methylguanine-DNA methyltransferase (*MGMT*) methylation status, extent of tumor resection (EOR), as well as the effects of sex and subtype on OS. Initially, we conducted univariate Cox regression analyses on each factor independently to identify those significantly associated with OS. Then, we performed multivariate Cox regression on the identified significant factors to explore their joint impact on OS.

Table 2 summarizes the Cox regression analyses in our study. We found that age, *MGMT* methylation, EOR, and subtype were significant factors associated with OS. According to the multivariate Cox regression results, younger age was associated with better survival (HR = 0.653, 95% CI 0.541–0.787; *p* < 0.05), whereas *MGMT* unmethylation was associated with poorer OS (HR = 1.508, 95% CI 1.140–1.995; *p* < 0.05) and partial resection was associated with worse survival (HR = 1.357, 95% CI 1.105–1.667; *p* < 0.05) as well. Subtype 3 had the best OS with HR = 0.586 (95% CI 0.469–0.732; *p* < 0.05), followed by subtype 2 with HR = 0.725 (95% CI 0.576–0.912; *p* < 0.05) and then subtype 1 had the worst OS. Furthermore, our analysis revealed that sex was not a significant factor with *p* > 0.05 in the univariate Cox regression.

**Table 2:** Univariate and multivariate Cox regression analyses for OS.

Factors	Univariate analysis HR (95% CI)	*p* value	Multivariate analysis HR (95% CI)	*p* value
Age				
> 65	(reference)		(reference)	
≤ 65	0.662 (0.551–0.794)	9.15E-06	0.653 (0.541–0.787)	8.09E-06
Sex				
Male	(reference)			
Female	0.976 (0.809–1.176)	0.795		
*MGMT* methylation				
Methylated	(reference)		(reference)	
Unmethylated	1.351 (1.027–1.777)	0.032	1.508 (1.140–1.995)	0.004
EOR				
Near/Gross total resection	(reference)		(reference)	
Partial resection or biopsy	1.503 (1.234–1.829)	4.94E-05	1.357 (1.105–1.667)	0.004
Subtype				
Subtype 1	(reference)		(reference)	
Subtype 2	0.729 (0.581–0.914)	0.006	0.725 (0.576–0.912)	0.006
Subtype 3	0.585 (0.469–0.729)	1.88E-06	0.586 (0.469–0.732)	2.47E-06

### Characterization of subtypes based on imaging and genomic data

#### Imaging data

As described in the "Feature selection and discovery-replication split" section, we utilized a feature selection process, resulting in the selection of 12 most relevant imaging features associated with the specific pathways involved in the genomic data. The details of selected features are listed in Table S1 in the supplementary material.

**Figure 3:**
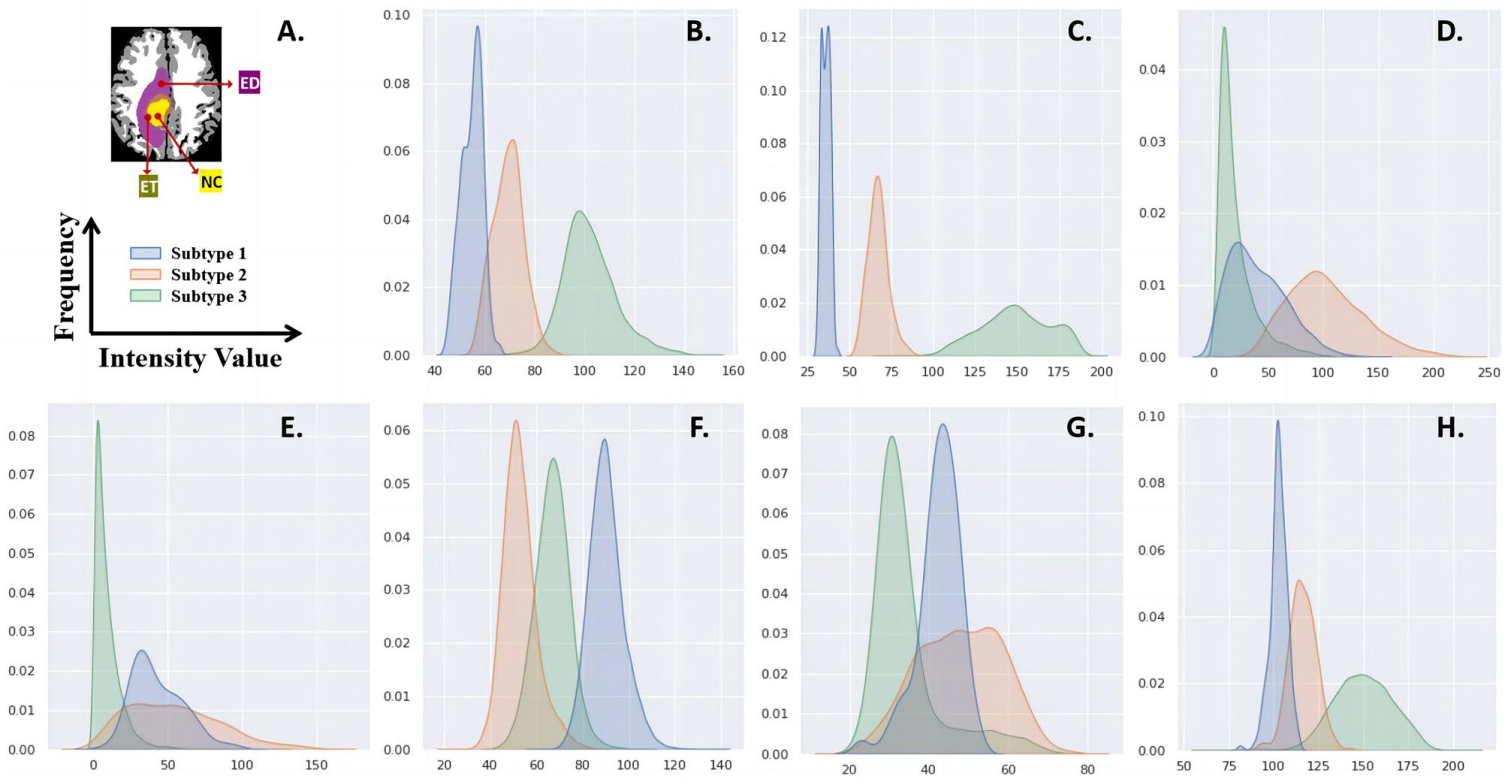
Histograms of intensity distribution in representative imaging characteristics in tumor subregions. (**A**) legends; (**B**) AD in ET; (**C**) RD in NC; (**D**) ap-rCBV in ET; (**E**) ap-rCBV in NC; (**F**) T1-subtraction in ED; (**G**) T1-Gd in NC; (**H**) T2 in NC.

Figure 3 illustrates the distribution of signal intensities within tumor subregions across the three subtypes, for the imaging scans from which the features were selected (AD = Axial diffusivity, RD = Radial diffusivity, ap-rCBV = automatically-extracted proxy to relative Cerebral Blood Volume, T1-subtraction = Subtraction of T1 from T1-Gd). The x-axis in our histogram represents the range of intensity values from 0 (black) to 255 (white). The y-axis denotes the frequency with which each intensity value occurs.

As indicated in the histograms for AD within ET and RD within NC (Fig. 3B,C), subtype 1 displayed the lowest average values with the smallest range of values, followed by subtype 2 with medium values, and subtype 3 with the highest value and the largest range of values. The distribution of ap-rCBV values within ET and NC as shown in Fig. 3D,E suggested lower cerebral perfusion within these regions for subtype 3, compared to the other subtypes. T1-subtraction in peritumoral edema region (Fig. 3F) showed more pixels around the higher range for subtype 1, compared to subtype 2 and 3, which may suggest more microvascular damage in ED in this group of tumors. Furthermore, lower range of T1-subtraction values in subtype 2 may indicate less microvascular leakage within the peritumoral area in this group. Taken together with concentration of normalized T1-Gd intensity values within NC towards lower range, in contrast to the other two subtypes in Fig. 3G, suggestive of lower microvascular leakage, it may be concluded that subtype 3 tumors may have lower neo-angiogenesis and disruption in microvascular architecture. As shown in Fig. 3H, subtype 1 tumors apparently had the most pixels with lower T2 values in NC, water concentration may be lower than other subtypes.

#### Genomic data

**Figure 4:**
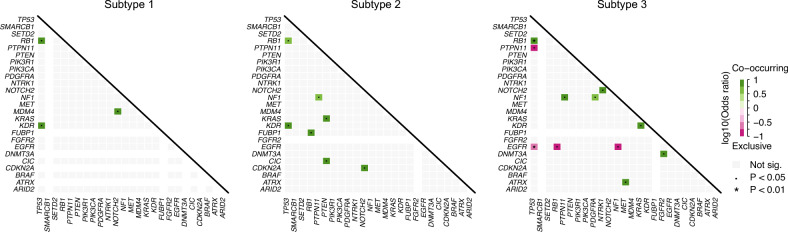
Heatmap of observed pairwise mutation patterns (odds ratio). Green colors denote preferential co-mutation, while pink colors indicate mutual exclusivity. (Fisher's exact test: *p* < 0.01, “*”;*p* < 0.05, “.”).

Figure 4 depicts the results of gene mutation co-occurrence and mutual exclusivity analyses on all 25 genes for different subtypes. For subtype 1, there were three pairs of genes with significant co-occurrence mutation patterns, which were [*TP53*,*RB1*], [*TP53*,*KDR*], and [*NOTCH2*,*MDM4*]. For subtype 2, there were seven pairs of genes with significant co-occurrence mutation patterns, which were [*TP53*,*RB1*], [*TP53*,*KDR*], [*RB1*,*FUBP1*], [*PTPN11*,*NF1*], [*PTEN*,*CIC*], [*PTEN*,*KRAS*], and [*NOTCH2*,*CDKN2A*]. For subtype 3, there were seven pairs of genes with significant co-occurrence mutation patterns, which were [*TP53*,*RB1*], [*PTPN11*,*NF1*], [*PDGFRA*,*NF1*], [*NTRK1*,*NOTCH2*], [*MET*,ATRX], [*KRAS*,*KDR*], and [*FGFR2*,*DNMT3A*]. Meanwhile, subtype 3 had four pair of genes with significant mutual exclusivity, which were [*TP53*,*PTPN11*], [*TP53*,*EGFR*], [*EGFR*,*RB1*], and [*EGFR*,*NF1*]. This indicates that if it has *TP53* mutations, it does not have several other mutations (*PTPN11*, *EGFR*), and if it has *EGFR* mutations, it does not have several other mutations (*TP53*, *RB1*, *NF1*).

Specifically, the gene pair [*TP53*,*PTPN11*] was found to be significantly co-occurrent in all three subtypes. In addition, the gene pair [*TP53*,*KDR*] was significantly co-occurrent in subtypes 1 and 2, while the pair [*PTPN11*,*NF1*] was significantly co-occurrent in subtypes 2 and 3. These findings suggested potential underlying biological mechanisms and pathways associated with the development and progression of glioblastoma across different subtypes.

Furthermore, as observed from Fig. S4 in our supplementary material, which illustrated the proportion of gene mutants within each subtype, it is evident that subtype 2 generally had fewer mutations. In contrast, subtypes 1 and 3 exhibited relatively larger mutation loads, particularly in genes like *TP53*, *PTEN*, *NF1*, and *EGFR*.

#### Imaging data vs. Genomic data

**Figure 5:**
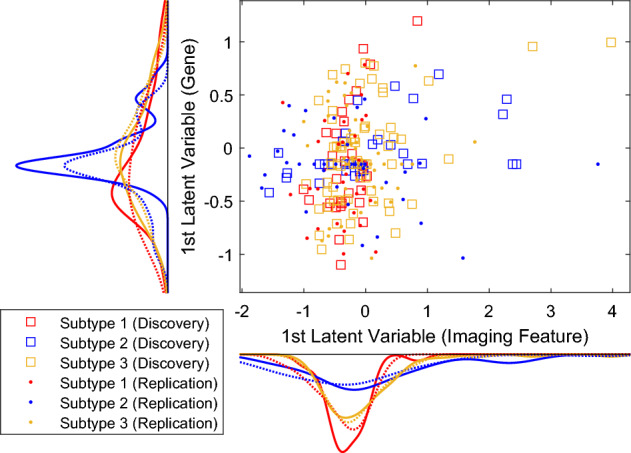
First latent variable from X (imaging features) vs. First latent variable from Y (genomics) in CCA. One point represents one glioblastoma subject. Subjects are colored according to their subtypes.

CCA found a pair of latent variables from two modalities. The correlation coefficient r = 0.3760 (*p* = 1.12E-9) of two 1st latent variables for the whole cohort, and r = 0.7002 (*p* = 4.75E-3) for discovery cohort, where r = 0.5124 (*p* = 3.20E-3) for subtype 1, r = 0.7952 (*p* = 1.93E-8) for subtype 2, r = 0.4269 (*p* = 3.51E-4) for subtype 3, respectively. Although the latent variables extracted by CCA were mixed up in the 2-D plot, their distributions among different subtypes were relatively different, as shown in Fig. 5. Additionally, we used the discovery cohort as a training set to predict the subtype of replication cohort in the latent variable space of CCA. A cross-validated k-nearest-neighbors (kNN) classifier with k = 13 achieved an accuracy of 53.04%. The classifier's ability to detect subtype 3 subjects with high accuracy was evident as 41 out of 46 subtype 3 subjects in the replication cohort were correctly predicted, representing 89.1% accuracy. The complete confusion matrix can be found in the supplementary material.

**Figure 6:**
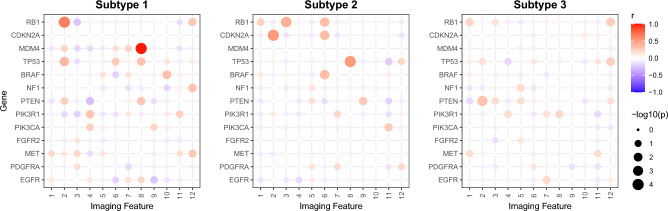
Bubble chart of correlation matrix between the variables of X (imaging features) and Y (genomics).

The heatmaps of the correlation matrices between imaging features and genomics, are presented in Fig. 6, indicating different correlations across different subtypes. Notably, subtype 1 was characterized by two significant correlated variable pairs: [imaging feature 8, *MDM4* gene] and [imaging feature 2, *RB1* gene]. For subtype 2, two significant correlated variable pairs were observed, *i.e.*, [imaging feature 8, *TP53* gene] and [imaging feature 2, *CDKN2A* gene]. Subtype 3 showed one significant correlated variable pair, *i.e.*, [imaging feature 2, *PTEN* gene]. These findings provided insights into the underlying relationships between imaging features and genomics in different subtypes. Note that *MDM4* and *TP53* were both part of the *P53* pathway, while *RB1* and *CDKN2A* belonged to the *RB1* pathway. Therefore, subtypes 1 and 2 exhibited similar correlations between imaging and genomic data due to the common involvement of these pathways, while subtype 3 displayed a distinct pattern of correlation.

## Discussion

This study developed an unsupervised multi-modal machine learning approach for glioblastoma subtype discovery. We used imaging features of mpMRI scans and genomic data to jointly identify three distinct glioblastoma subtypes and discussed their clinical, imaging, and genomic characteristics. We also performed canonical correlation analysis between imaging and genomic data for each subtype and identified significant correlations that could potentially provide insights into the underlying biology of the disease.

*Significant prognostic factors*. In the present study, survival analysis utilizing Cox regression reinforced the findings of prior studies^11,32–35^ about the significant factors associated with better OS, including younger age, *MGMT* methylation, and near/gross total resection. However, contrary to some prior research^36–42^, sex was not a significant factor in the UPenn-GBM cohort based on our analysis. Previous research indicated that glioblastoma incidence is higher among males^36–40^ and female patients generally have better outcomes^36,40–42^.

*Significant imaging features of DTI-MRI*. For the RD and AD characteristics, a lower intensity value suggests that the tumor cells in those areas are more compact and densely packed, which is generally indicative of higher tumor cellularity and aggressiveness. Tumors that exhibit higher cellularity and aggressiveness tend to be more invasive, meaning that they are more likely to grow into nearby tissue, and are often more resistant to treatment. This can lead to a poorer prognosis for patients with these types of tumors. A smaller range indicates that the tumor cells are more homogenous in this area. This could be an indication of a more uniform composition of tumor cells in that area, rather than a mixture of different cell types. A larger range is compatible with greater cellular heterogeneity, either among neoplastic cells, or also considering other lineages such as vascular and hematopoietic cells.

Based on our findings, subtype 1 with the worst OS in our cohort has the lowest RD and AD compared with other subtypes. This may indicate more severe damage to the tissue, such as increased cellularity or necrosis in both enhancing and non-enhancing areas. Lower AD and RD values in subtype 1 may suggest higher microstructural damage in the tissue, compared to subtypes 2 and 3. As subtype 1 tumors apparently have more pixels with lower T2 values in the non-enhancing core, water concentration may be lower in this subtype, with along with lower RD, it may infer that the tumors in this group have more microstructural disruption.

*Co-occurrence of genes.* Although we analyzed only a subset of the molecular changes known to be important in glioblastoma, the results found several significant associations. The gene pair [*TP53*,*PTPN11*] significantly co-occurred in all three subtypes. The co-occurrence of *TP53* and *PTPN11* gene mutations in glioblastoma suggests that these genes may work together to drive the development and progression of the disease. *TP53* mutations may lead to the loss of its tumor suppressor function, allowing cancer cells to grow and divide uncontrollably^43^. Meanwhile, *PTPN11* mutations may activate signaling pathways that promote cell proliferation and survival^44^. The co-occurrent mutations may suggest aggressive growth and poor prognosis of glioblastoma.

In addition, the gene pair [*TP53*,*KDR*] was significantly co-occurrent in subtypes 1 and 2, while the pair [*PTPN11*,*NF1*] was significantly co-occurrent in subtypes 2 and 3. This observation suggests that subtype 2 may serve as a transitional or intermediate stage between subtype 1 and subtype 3. *KDR* is a gene that encodes a protein called VEGFR2, which is a receptor for vascular endothelial growth factor (VEGF)^45^. This receptor plays a key role in regulating angiogenesis, the process by which new blood vessels are formed. Angiogenesis is critical for tumor growth and metastasis, as it provides nutrients and oxygen to the tumor and allows cancer cells to spread to other parts of the body. The co-occurrence of *TP53* and *KDR* mutations may work together to promote angiogenesis, allowing the tumor to grow and spread more aggressively. *NF1* is a tumor suppressor gene that helps regulate cell division and prevent tumor formation^46^. The co-occurrence of *PTPN11* and *NF1* mutations may work together to promote the uncontrolled growth and proliferation of cancer cells in glioblastoma.

Comparing the distinct OS outcomes of subtype 1 and 3, and taking into account the proportion of gene mutants and the co-occurrence of mutations, it appears that the co-occurrence of mutations may give rise to imaging patterns (tumor phenoptypes) with poor survival. Subtype 3 with good survival had the most significant mutual exclusivity, while subtype 1 with poor survival had no mutual exclusivity. This suggests that one needed to hit multiple pathways to achieve the most aggressive phenotype.

*Correlation between imaging and genomic data.* From our analysis on subtype 1 and 2, the imaging feature 2 ('RD_NC_Histogram_Bins-16_Bins-16_Bin-0_Probability') is positively correlated to *RB1* gene pathway. RD values reflect the degree of water diffusion perpendicular to the primary direction of the white matter fibers in the brain. The RD histogram feature could potentially be used as a quantitative imaging biomarker to assess the degree of tissue disruption, cellularity, and/or edema within the non-enhancing core of a glioblastoma. Therefore, an increase in activity in the *RB1* pathway may correspond to a positive change in the RD histogram feature, indicating a decrease in tissue organization or an increase in cellularity. Similar relations can be drawn from subtype 3 with *PTEN* gene.

*Clinical implications of the GBM subtypes.* The main challenge in caring for patients with GBM is the dearth of effective therapies. However, when using the treatments with proven survival benefits, including temozolomide, radiation, and tumor treating fields, progression-free survival and overall survival can vary widely across patients and cannot be reliably predicted by the usual prognostic features alone (age, *MGMT* methylation status, extent of resection, and performance status). Our integrated imaging and genomic signature for GBM subtyping may provide more accurate prognostication for individual patients, leading to more personalized and overall better care as well as a useful tool for risk stratification in the context of clinical trials. In addition, the field is actively developing new targeted therapies, which are being tested in clinical trials. Insight from our GBM analysis on prognosis and biological features of the tumor may aid in patient selection for these studies. Moreover, we have shown in a previous publication^47^ that having a good predictor of outcome can significantly boost the power of clinical trials to detect a treatment effect, as it allows us for patient stratification and for normalization of outcome measures by expected outcome measures. As these predictors improve, the gain in power will increase. Conversely, fewer patients are needed for a clinical trial to detect treatment effects.

*Generalizability of the algorithm.* Glioma diagnoses depend on molecular features, and therefore, the molecular features are different when considering GBM, which are IDH-wildtype by definition, and other infiltrating gliomas, such as IDH-mutant astrocytomas. Although the subtypes discovered by the algorithm working on the radiological imaging and sequencing data from GBMs are not necessarily relevant to other gliomas, the framework can be utilized to elucidate the relationships of molecular and imaging features of these other gliomas, and potentially to aid in prognostication, as in the case of GBM.

*Limitations.* One of the main limitations of our study is that we analyzed a small sample size from a single institution. As a result, we had a limited number of patients with imaging features and/or genomics within each subtype, which prompted us to analyze the histogram, co-occurrence, and correlation on the entire cohort, without using the discovery and replication split setting. To validate our results, further studies are required on larger scale, multi-institutional studies such as the ReSPOND consortium^48^. In addition, we plan to extend and refine our findings through a larger sequencing panel that will capture additional targets, copy number changes, and fusions, as well as by incorporating methylation profiling. Finally, our intention in this paper was not only to provide insights into the synergistic value of radiomic and molecular characteristics, but also to lay the groundwork for subsequent investigations into the nonlinear aspects of radiomic-genomic relationships. We aim to build upon the foundations established in this pioneering work and delve deeper into the complexities of nonlinear relationships between radiomic and genomic date in future studies.

*Summary.* This study takes a distinctive approach to analyzing glioblastoma by utilizing data-driven unsupervised multi-modal analyses to investigate its phenotypical and molecular heterogeneity. Unlike previous studies that relied on either imaging features or genomics to make modality-specific predictions, this work aims to uncover hidden patterns and structures across multiple data modalities, without any prior knowledge or preconceived target of clinical characteristics. Joint learning on both imaging and genomic data is particularly beneficial in understanding the complex interplay between different aspects of glioblastoma, ultimately provides insights into the biologic underpinnings of tumor formation and progression.

### Supplementary Information

Supplementary Information.

## Data Availability

The datasets generated during and/or analyzed during the current study are available from the corresponding author on reasonable request.
