# Supplementary material for: Integrating imaging and genomic data for the discovery of distinct glioblastoma subtypes: a joint learning approach
Source: Sci Rep. 2024 Feb 28;14:4922. doi: 10.1038/s41598-024-55072-y (PMC10902376; doi:10.1038/s41598-024-55072-y)
Supplement: Supplementary file 1 — Supplementary Information. [file 41598_2024_55072_MOESM1_ESM.docx]

**Integrating Imaging and Genomic Data for the Discovery of Distinct Glioblastoma Subtypes: A Joint Learning Approach**

Jun Guo^1,2,3^, Anahita Fathi Kazerooni^1,2,4,5^, Erik Toorens^6^, Hamed Akbari^1,2,3,7^, Fanyang Yu^1,2^, Chiharu Sako^1,2,3^, Elizabeth Mamourian^1,3^, Russell T. Shinohara^1,8^, Constantinos Koumenis^9^, Stephen J. Bagley^10,11^, Jennifer J. D. Morrissette^12^, Zev A. Binder^5,11^, Steven Brem^5,11^, Suyash Mohan^1,2,3^, Robert A. Lustig^9,10^, Donald M. O’Rourke^5,11^, Tapan Ganguly^6,10^, Spyridon Bakas^1,2,^^3,12,13^, MacLean P. Nasrallah^1,2,12,#^, Christos Davatzikos^1,2,3,#,*^

^1^ Center for Biomedical Image Computing and Analytics (CBICA), University of Pennsylvania, 3700 Hamilton Walk, 7th floor, Philadelphia, PA, 19104, USA.

^2^ Center for AI and Data Science for Integrated Diagnostics, University of Pennsylvania, Philadelphia, USA.

^3^ Department of Radiology, Perelman School of Medicine, University of Pennsylvania, Philadelphia, PA, USA.

^4^ Center for Data-Driven Discovery in Biomedicine (D^3^b), Division of Neurosurgery, Children’s Hospital of Philadelphia, Philadelphia, PA, USA.

^5^ Department of Neurosurgery, Perelman School of Medicine, University of Pennsylvania, Philadelphia, PA, USA.

^6^ Penn Genomic Analysis Core, Perelman School of Medicine, University of Pennsylvania, Philadelphia, PA, USA.

^7^ Department of Bioengineering, School of Engineering, Santa Clara University, Santa Clara, CA, USA.

^8^ Penn Statistics in Imaging and Visualization (PennSIVE) Center, Department of Biostatistics, Epidemiology, and Informatics, Perelman School of Medicine, University of Pennsylvania, Philadelphia, PA, USA.

^9^ Department of Radiation Oncology, Perelman School of Medicine, University of Pennsylvania, Philadelphia, PA, USA.

^10^ Abramson Cancer Center, Perelman School of Medicine, University of Pennsylvania, Philadelphia, PA, USA.

^11^ Glioblastoma Translational Center of Excellence, Abramson Cancer Center, University of Pennsylvania, Philadelphia, PA, USA.

^12^ Department of Pathology & Laboratory Medicine, Perelman School of Medicine, University of Pennsylvania, Philadelphia, PA, USA.

^13^ Division of Computational Pathology, Department of Pathology & Laboratory Medicine, School of Medicine, Indiana University, Indianapolis, IN, USA.

^#^ Equally contributing senior authors

^*^ Correspondence: [Christos.Davatzikos@pennmedicine.upenn.edu](mailto:Christos.Davatzikos@pennmedicine.upenn.edu)

Supplementary tables

Table S1: Description of the 12 selected radiomic features. AD = Axial diffusivity, RD = Radial diffusivity, ap-rCBV = automatically-extracted proxy to relative Cerebral Blood Volume, ET = enhancing tumor, NC = Non-enhancing core, ED = Edema.

|  | **Feature name** | **Feature description** |
| --- | --- | --- |
| 1 | AD_ET_Histogram_Bin-2_Frequency | This feature measures the frequency of axial diffusivity (AD) values in the enhancing tumor (ET) region, with a histogram divided into 16 bins and the second bin selected for analysis. |
| 2 | RD_NC_Histogram_Bin-0_Probability | This feature measures the probability of radial diffusivity (RD) values in the non-enhancing core (NC) region, with a histogram divided into 16 bins and the first bin selected for analysis. |
| 3 | RD_NC_Histogram_Bin-4_Probability | This feature measures the probability of radial diffusivity (RD) values in the non-enhancing core (NC) region, with a histogram divided into 16 bins and the fifth bin selected for analysis. |
| 4 | RD_NC_Histogram_Bin-10_Probability | This feature measures the probability of radial diffusivity (RD) values in the non-enhancing core (NC) region, with a histogram divided into 16 bins and the eleventh bin selected for analysis. |
| 5 | ap-RCBV_ET_Intensity_Minimum | This feature measures the minimum intensity of ap-rCBV images in the enhancing tumor (ET) region. |
| 6 | ap-RCBV_ET_Histogram_QuartileCoefficientOfVariation | This feature measures the coefficient of variation (a measure of variability) of ap-rCBV image intensities in the enhancing tumor (ET) region, with a histogram divided into 16 bins and the quartile selected for analysis. |
| 7 | ap-RCBV_NC_Histogram_Bin-4_Probability | This feature measures the probability of ap-rCBV image intensities in the non-enhancing core (NC) region, with a histogram divided into 16 bins and the fifth bin selected for analysis. |
| 8 | ap-RCBV_NC_Histogram_Bin-14_Probability | This feature measures the probability of ap-rCBV image intensities in the non-enhancing core (NC) region, with a histogram divided into 16 bins and the fifteenth bin selected for analysis. |
| 9 | T1_ED_Histogram_Bin-0_Probability | This feature measures the probability of T1-weighted enhancement values in the edema (ED) region, with a histogram divided into 16 bins and the first bin selected for analysis. |
| 10 | T1Gd_ED_Histogram_Bin-15_Probability | This feature measures the probability of T1-Gd in the edema (ED) region, with a histogram divided into 16 bins and the sixteenth bin selected for analysis. |
| 11 | T1Gd_NC_Histogram_Bin-0_Frequency | This feature measures the frequency of T1-Gd values in the non-enhancing core (NC) region, with a histogram divided into 16 bins and the first bin selected for analysis. |
| 12 | T2_NC_Histogram_Bin-3_Probability | This feature measures the probability of T2-weighted values in the non-enhancing core (NC) region, with a histogram divided into 16 bins and the fourth bin selected for analysis. |

Table S2: Univariate and multivariate Cox regression analyses for OS on discovery and replication cohorts. Some results are not significant anymore due to the limited patients after the discovery-replication split.

| **Factors** | **Univariate analysis**  **HR (95% CI)** | **P-value** | **Multivariate analysis**  **HR (95% CI)** | **P-value** |
| --- | --- | --- | --- | --- |
| **Discovery cohort** | | | | |
| **Age** |  |  |  |  |
| > 65 | (reference) |  | (reference) |  |
| ≤ 65 | 0.727 (0.554-0.954) | 0.022 | 0.734 (0.552-0.976) | 0.034 |
| **Sex** |  |  |  |  |
| Male | (reference) |  |  |  |
| Female | 1.044 (0.791-1.377) | 0.762 |  |  |
| ***MGMT* methylation** |  |  |  |  |
| Methylated | (reference) |  | (reference) |  |
| Unmethylated | 1.601 (1.083-2.366) | 0.018 | 1.733 (1.130-2.657) | 0.012 |
| **EOR** |  |  |  |  |
| Near/Gross total resection | (reference) |  | (reference) |  |
| Partial resection or biopsy | 1.627 (1.218-2.175) | 0.001 | 1.581 (1.156-2.162) | 0.004 |
| **Subtype** |  |  |  |  |
| Subtype 1 | (reference) |  | (reference) |  |
| Subtype 2 | 0.690 (0.492-0.966) | 0.031 | 0.758 (0.530-1.085) | 0.130 |
| Subtype 3 | 0.544 (0.392-0.755) | 2.67E-04 | 0.557 (0.399-0.778) | 5.86E-04 |
| **Replication cohort** | | | | |
| **Age** |  |  |  |  |
| > 65 | (reference) |  | (reference) |  |
| ≤ 65 | 0.597 (0.465-0.767) | 5.56E-05 | 0.582 (0.450-0.753) | 3.68E-05 |
| **Sex** |  |  |  |  |
| Male | (reference) |  |  |  |
| Female | 0.936 (0.726-1.206) | 0.608 |  |  |
| ***MGMT* methylation** |  |  |  |  |
| Methylated | (reference) |  |  |  |
| Unmethylated | 1.129 (0.767-1.664) | 0.538 |  |  |
| **EOR** |  |  |  |  |
| Near/Gross total resection | (reference) |  | (reference) |  |
| Partial resection or biopsy | 1.430 (1.092-1.873) | 0.009 | 1.220 (0.925-1.609) | 0.159 |
| **Subtype** |  |  |  |  |
| Subtype 1 | (reference) |  | (reference) |  |
| Subtype 2 | 0.742 (0.546-1.009) | 0.057 | 0.716 (0.526-0.975) | 0.034 |
| Subtype 3 | 0.614 (0.455-0.830) | 0.002 | 0.584 (0.429-0.795) | 6.21E-04 |

**Table S3**: Mean and median survival months of three subtypes.

|  | Subtype 1 | Subtype 2 | Subtype 3 |
| --- | --- | --- | --- |
| Mean | 11.32 (Discovery cohort: 11.34; Replication cohort: 11.31) | 14.85 (Discovery cohort: 15.56; Replication cohort: 14.24) | 18.09 (Discovery cohort: 19.87; Replication cohort: 16.54) |
| Median | 9.47 (Discovery cohort: 9.47; Replication cohort: 9.17) | 12.63 (Discovery cohort: 12.58; Replication cohort: 12.93) | 14.17 (Discovery cohort: 14.57; Replication cohort: 13.55) |

Supplementary figures


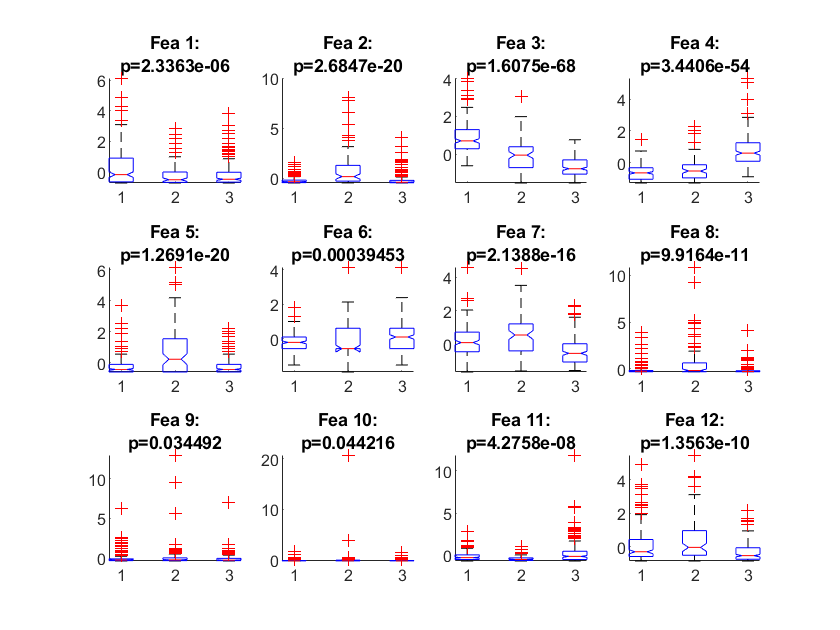


Figure S1. Image features significantly associated with each subtype calculated via ANOVA for the whole cohort. The x-axis represents subtype 1, 2, and 3. The y-axis denotes the value of imaging features.


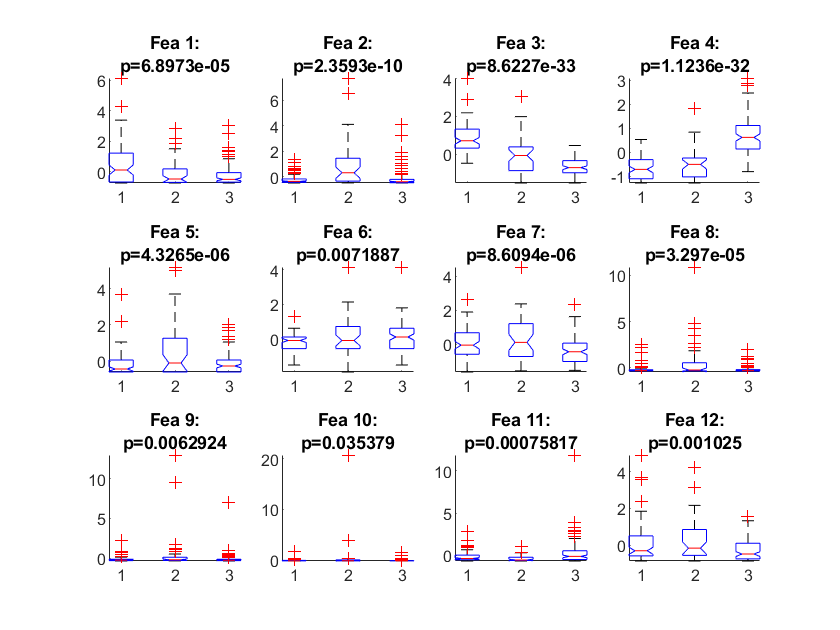


Figure S2. Image features significantly associated with each subtype calculated via ANOVA for the discovery cohort. The x-axis represents subtype 1, 2, and 3. The y-axis denotes the value of imaging features.


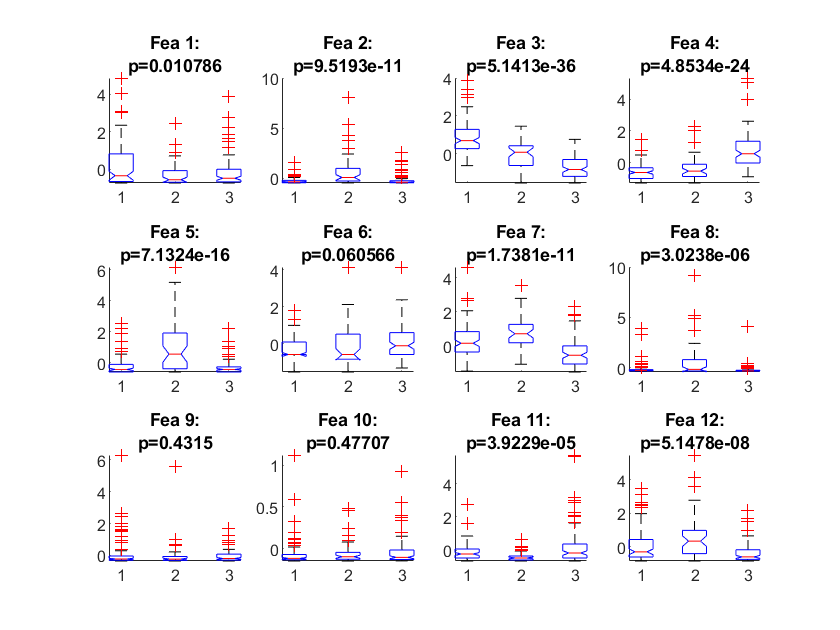


Figure S3. Image features significantly associated with each subtype calculated via ANOVA for the replication cohort. The x-axis represents subtype 1, 2, and 3. The y-axis denotes the value of imaging features.


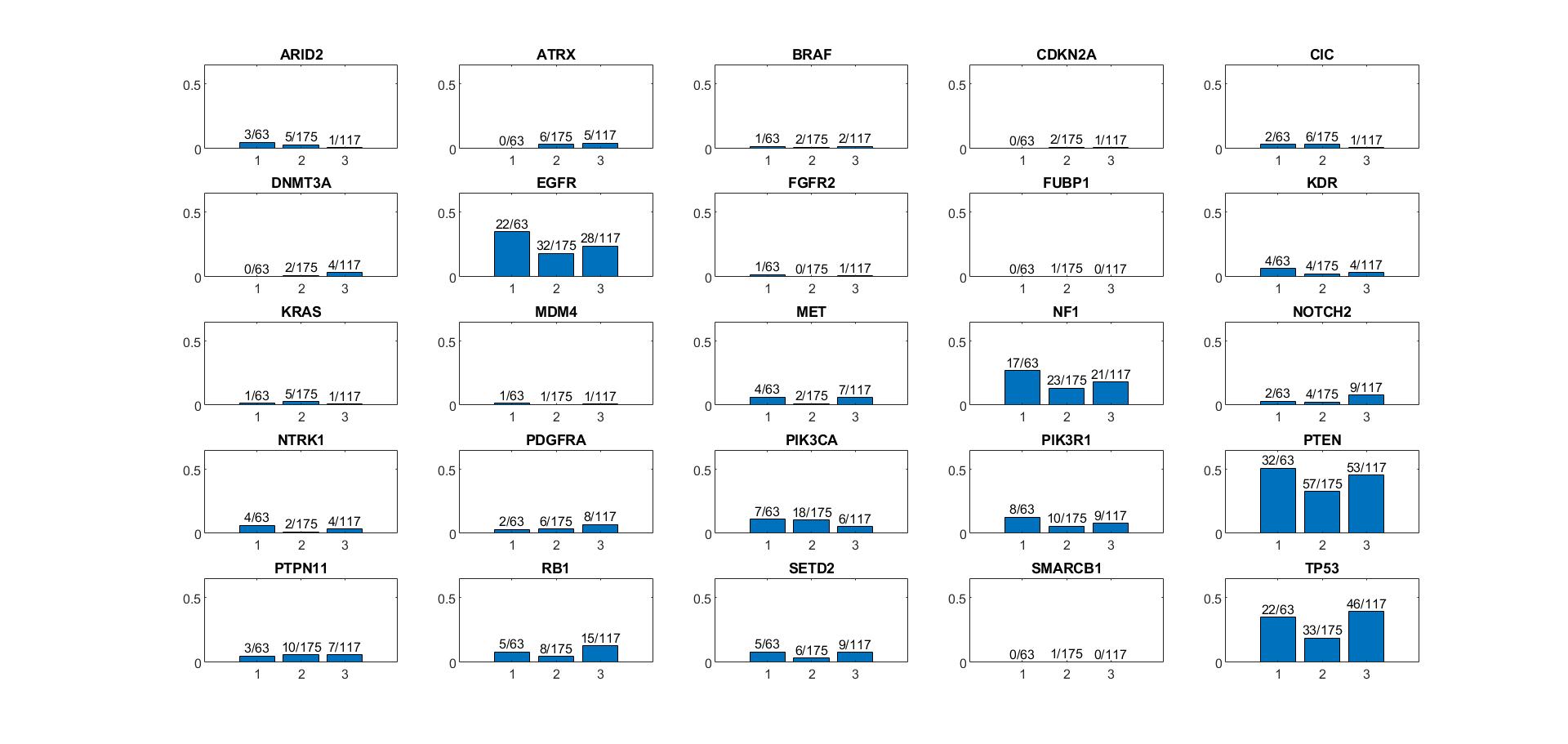


Figure S4. Proportion of mutants (Y-axis) within each subtype (X-axis) for the whole cohort.


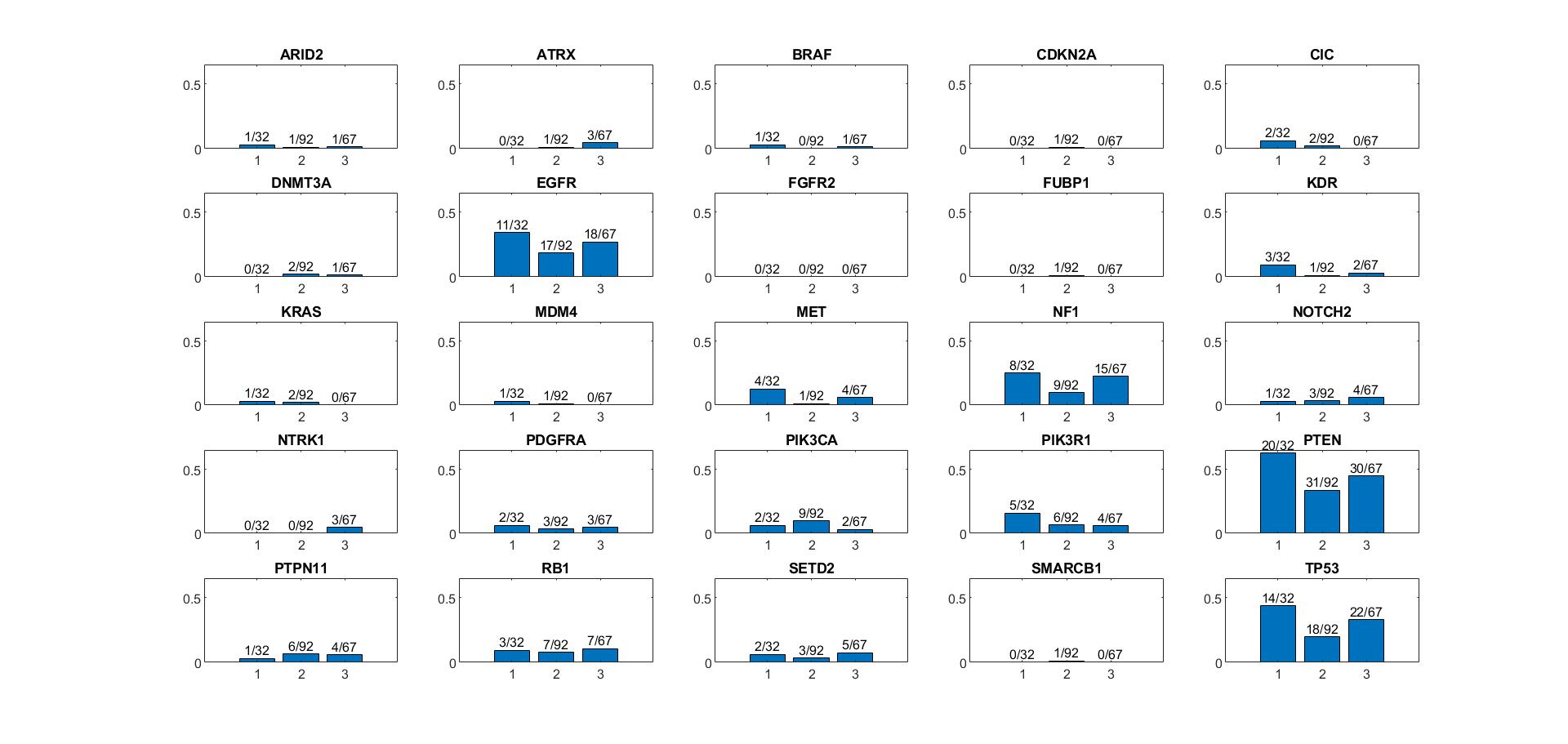


Figure S5. Proportion of mutants (Y-axis) within each subtype (X-axis) for the discovery cohort.


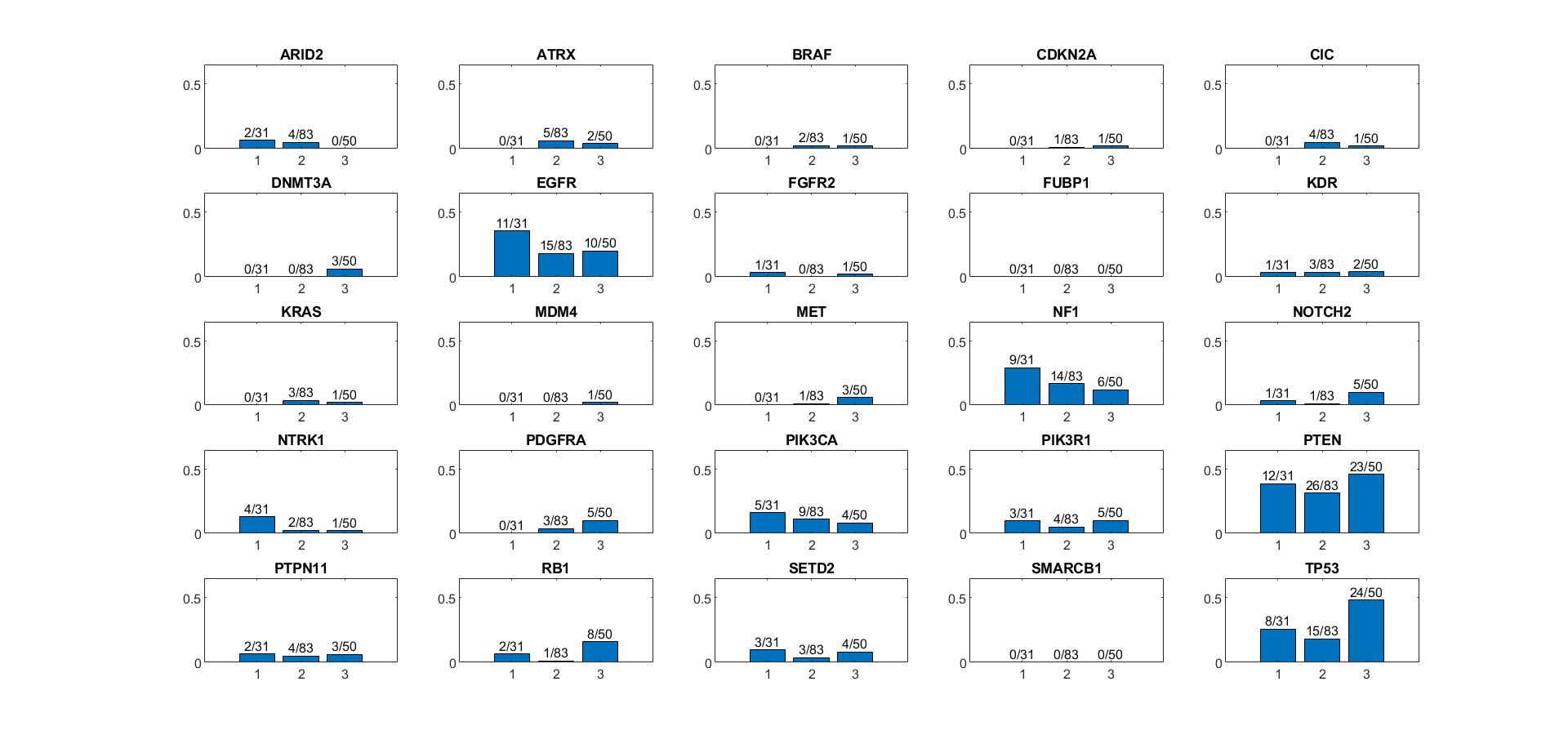


Figure S6. Proportion of mutants (Y-axis) within each subtype (X-axis) for the replication cohort.


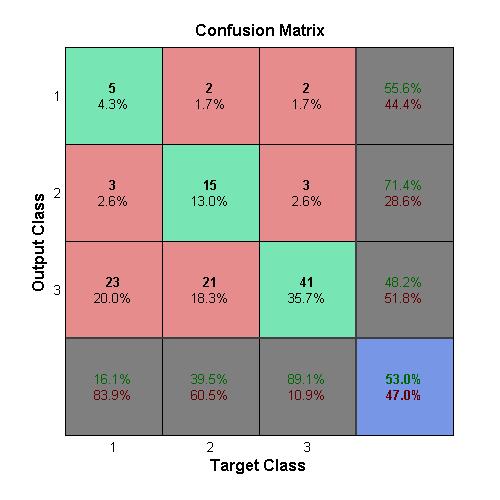


Figure S7. **The confusion matrix of kNN classifier with k = 13 when using the discovery cohort as training set to predict the subtype of replication cohort in the latent variable space of CCA.** In this confusion matrix, the first three diagonal cells show the number and percentage of correct classifications by the trained classifier. For example, 5 subjects are correctly classified as subtype 1. This corresponds to 4.3% of all 115 subjects in the discovery cohort. Similarly, 15 cases are correctly classified as subtype 2 and this corresponds to 13.0% of all 115 subjects. Besides, 41 subjects correctly classified as subtype 3 and this corresponds to 35.7% of all 115 subjects. Considering each row, out of 9 predictions with subtype 1 as output, 55.6% are correct and 44.4% are wrong. Out of 21 predictions with subtype 2 as output, 71.4% are correct and 28.6% are wrong. Out of 85 predictions with subtype 3 as output, 48.2% are correct and 51.8% are wrong. Considering each column, out of 31 subtype 1 subjects, 5 cases (16.1%) are correctly predicted. Out of 38 subtype 2 subjects, 15 cases (39.5%) are correctly predicted. Out of 46 subtype 3 subjects, 41 cases (89.1%) are correctly predicted. Overall, 53.0% of the predictions are correct.


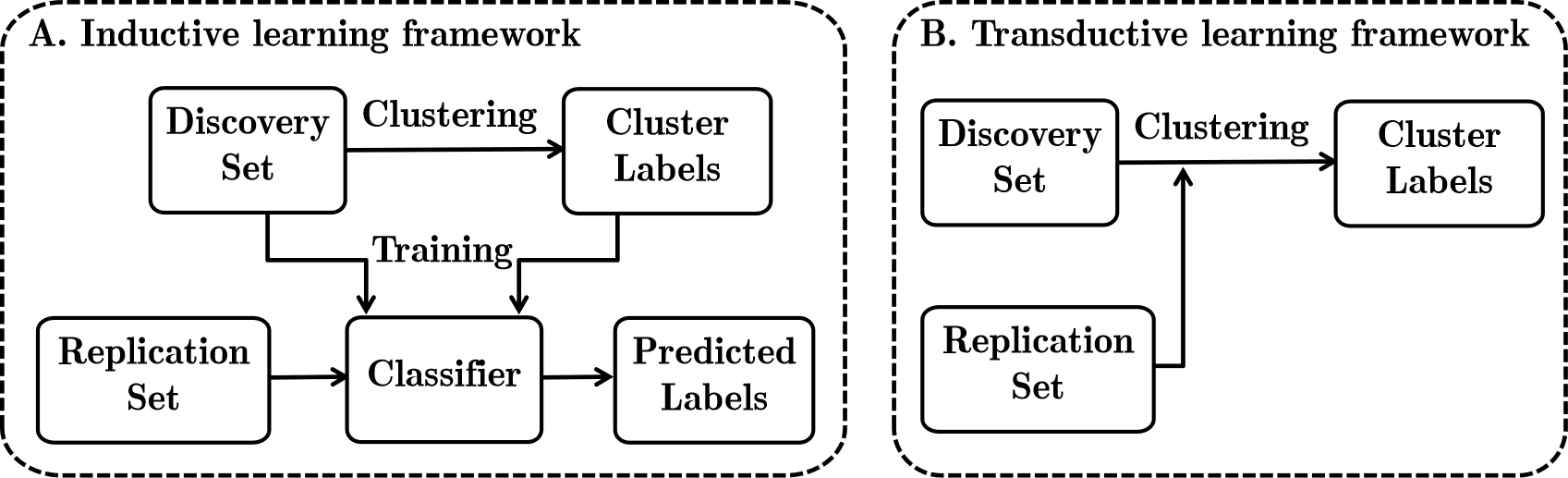


Figure S8. The comparison of frameworks in (A) traditional methods as an inductive framework and (B) our joint learning method as a transductive framework.
